# Do Coping Motives and Perceived Impaired Control Mediate the Indirect Links from Childhood Trauma Facets to Alcohol-Related Problems?

**DOI:** 10.3390/bs13030197

**Published:** 2023-02-23

**Authors:** Jai Bitsoih, Julie A. Patock-Peckham, Jessica R. Canning, Annie Ong, Allison Becerra, Matthew Broussard

**Affiliations:** 1 Department of Psychology, Arizona State University, Tempe, AZ 85287-1104, USA; 2 Department of Psychology, University of Washington, Seattle, WA 98195-1525, USA; 3 Department of Counseling, Loma Linda University, Loma Linda, CA 92350, USA

**Keywords:** childhood trauma, coping motives, impaired control over drinking, physical neglect, emotional abuse, sexual abuse

## Abstract

Introduction: The Self-Medication Hypothesis suggests that individuals drink to alleviate undesirable affective states. Behavioral Economics Theory states that individuals deprived of resources (i.e., physically neglected) consume more reinforcing substances when they are available than others. Childhood trauma may indirectly increase impaired control over alcohol (IC; drinking beyond one’s own intentions) and thereby increase alcohol use and problems through the employment of coping-motives. Method: A structural equation model that included sex as a covariate examined mediated paths with 612 university students. Results: Men were less likely to be emotionally abused and were more likely to use greater amounts of alcohol than women did. Physical neglect was directly linked to both more IC and alcohol use. Emotional and sexual abuse were directly linked to more coping motives. Both emotional and sexual abuse were indirectly linked to more alcohol use and its related problems through increased coping motives and IC. Conclusions: Consistent with Behavioral Economics Theory, there was a direct link between physical neglect and IC. We also found partial support for the Self-Medication Hypothesis regarding the emotional and sexual abuse trauma dimensions; they indirectly contributed to alcohol use and its related problems via the mediating mechanisms of more coping motives and IC. Our findings suggest coping motives could be a therapeutic target for intervention among those sexually or emotionally abused.

“*Trauma is a fact of life. It does not, however, have to be a life sentence*”.

[1]

## 1. Introduction

The Self-Medication Hypothesis (SMH) [2,3,4] posits that individuals drink to alleviate negative affective states. Victims of childhood trauma often experience negative affect, have greater difficulty coping with psychological distress [5] and thereby turn to dysregulated alcohol misuse [3,6,7]. This is important because approximately 618,000 children and infants were victims of childhood abuse and neglect in the United States in 2020 [8]. Thus, we hypothesize that these individuals will consume more alcohol to cope with the psychological symptoms linked to a childhood trauma history prior to the age of 12, as well as violate their intentions to limit alcohol consumption more regularly. Violating one’s intentions to consume less alcohol or drinking for longer periods than intended is impaired control over alcohol (IC) [9].

When children experience trauma during vulnerable and sensitive periods of development, they are at risk of a myriad of detrimental effects on their future physiological, behavioral, and emotional functioning. For instance, childhood maltreatment is associated with a wide range of physical health consequences, including diabetes, lung disease, arthritis, heart attack, high blood pressure, migraine headaches, bowel disease, stroke, and cancer later on in life [10,11,12,13]. Childhood trauma was also associated with risky behaviors, such as initiating sex at an earlier age, transactional sex, juvenile delinquency, and adult criminal behaviors [10]. A history of childhood trauma is associated with an increased risk of internalizing disorders [14], such as a lack of mindfulness [15], insomnia [16], post-traumatic stress disorder (PTSD) [6,17], as well as depression and anxiety [18]. 

It is well documented that childhood trauma negatively affects adult survivors’ emotional pathways to alcohol use disorders (AUDs) [12,19]. Using an overall composite variable including all 25 items of the Childhood Trauma Questionnaire (CTQ) [20], Shin et al. [21] found that coping motives for drinking did mediate the indirect link between overall childhood trauma and alcohol-related problems. Nevertheless, Shin et al. [21] failed to control for alcohol use quantity/frequency in their model and did not include a measure of dysregulated drinking, such as impaired control over alcohol, as another potential mediator of this relationship. According to Dawson [22], one important goal of alcohol epidemiology is to link alcohol consumption to alcohol-related problems; therefore, alcohol consumption must be determined and measured as accurately as possible. Measuring both alcohol use quantity/frequency and alcohol-related problems allows one to adjust for individual characteristics that may confound the associations studied. Keyes et al., [23] in a NESARC study of 43,093 adults over the age of 18, found that the inclusion of an alcohol use quantity/frequency measure eliminates false positives from studies of alcohol disorder etiology. For instance, several internalizing constructs are directly linked to increases in alcohol-related problems but not to alcohol use per se [24,25,26]. 

Coping motives [27] reflect drinking to forget one’s worries and problems in the hopes of elevating one’s mood; this is drinking because it helps you when you feel depressed or nervous or need a boost in self-confidence. Higher levels of coping motives have been directly linked to a perceived impaired control over alcohol (IC) within a model of perfectionism facets [28]. Coping motives were also indirectly linked to more alcohol use and alcohol-related problems [28]; both coping motives and IC mediate the relationship between perfectionism discrepancy and alcohol-related problems. Yet, these relationships concerning childhood trauma histories have been left woefully unexplored. Thus, we sought to examine the specific mechanisms involved. We tested when both coping motives and IC were used in conjunction within the same model while in the presence of a history of childhood trauma. Furthermore, rarely are investigators studying all facets of trauma at once within the same model. Often investigators only use a composite variable for trauma in their mediation models of alcohol use disorders [21]. Others draw overall conclusions with composites of childhood trauma/maltreatment [6,7,29,30], or they just sum up instances of trauma events [11].

Childhood trauma is a multifaceted construct consisting of various forms of neglect and abuse. Bernstein et al. [31] described childhood maltreatment as involving a number of different facets. For instance, physical neglect is the failure to meet basic physical needs such as food, shelter, or health care. Emotional abuse is undergoing demeaning or humiliating verbal assault. Sexual abuse is suffering inappropriate sexual contact such as touching or rape. Physical abuse is facing bodily assault such as being hit or slapped. Lastly, emotional neglect is having caregivers who fail to meet support, nurturance, or belonging needs. Of these, all specific categories of maltreatment have documented direct and indirect associations with later alcohol use [15,17,32,33,34,35]. Yet, to date, only a few studies have examined all the dimensions of trauma simultaneously [15,16,17]. Moreover, none of these recent aforementioned studies have examined coping motives (i.e., drinking because it helps you feel less depressed or nervous) as a mediating mechanism between trauma and impaired control over alcohol (IC; i.e., dysregulated alcohol consumption) along the pathways to alcohol use and alcohol-related problems.

### 1.1. The Self-Medication Hypotheses and Coping Motives 

**Figure 1:**
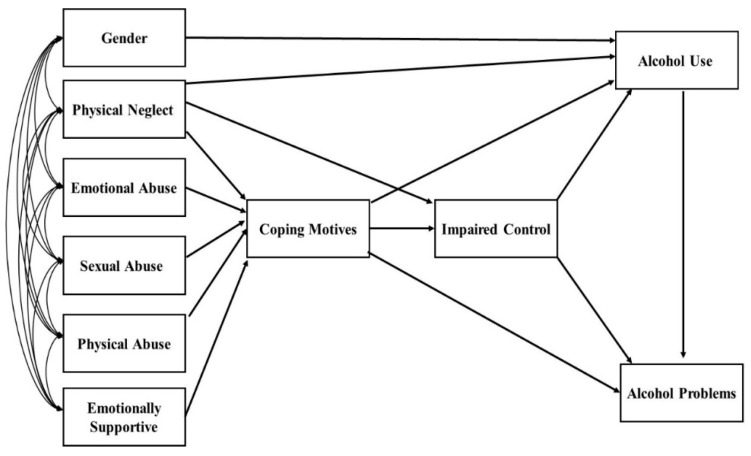
Conceptual model that allowed all the exogenous variables to correlate with each other. This model shows each path we tested for significance.

According to the Self-Medication Hypothesis (SMH) [2,3,4], people with higher coping motives for drinking should experience more alcohol-related problems. Cooper et al. [36] found coping motives to be significantly linked to alcohol-related problems, both directly and indirectly through alcohol use. In fact, coping motives are associated with greater alcohol-related problems even when controlling for the amount of alcohol consumed [37]. Furthermore, individuals who drink to cope were also more likely to engage in hazardous drinking behavior, such as drinking in solitary conditions [27] and heavy-drinking behaviors [38]. Figure 1 depicts all the variables and direct and indirect relationships we tested in our model. 

### 1.2. Trauma, PTSD, and Coping Motives for Drinking

Posttraumatic stress disorder (PTSD) is a cluster of symptoms (e.g., flashbacks, nightmares, severe anxiety, and avoidance of people or places) triggered by a terrible event, such as a traumatic experience, that threatened an individual in the past. While we are not measuring or studying PTSD in this study, some may consider PTSD to be a symptom of past trauma experience. As such, we will conduct a short review of this literature in this section. Both sexual and emotional abuse were found to be directly linked to more PTSD symptoms among university students even when including other forms of trauma such as physical abuse, a lack of support, and physical neglect in the same model [17]. This is important because Tomaka et al. [39] found that drinking to cope mediated PTSD symptoms in problematic drinking among 740 (98% male) municipal firefighters. Yet, Tuliao et al. [40] were unable to find a relationship between PTSD and alcohol use with tension reduction expectancies (i.e., I would feel calm; I would feel peaceful; my body would feel relaxed) [41] (p. 23); however, numerous others have found motives to mediate this relationship. Kaysen et al. [42] demonstrated that drinking to cope mediated the relationship between domestic violence and peak alcohol use, [ measured with Timeline Followback (TLFB) [43]], among 369 battered adult women. Betrayal trauma prior to the age of 18 was directly associated with PTSD and problematic substance use when using alcohol to cope; this was measured as a latent indicator of problematic use [44]. Enhancement and coping motives served as mediators of PTSD symptoms in alcohol consumption, whereas only coping motives mediated PTSD symptoms in alcohol-related problems among 509 middle-aged (38.7 mean years; 83% men) recent military service members [45]. Mohr et al. [46] examined drinking motives and hazardous drinking among current and former U.S. Service members. They found that enhancement (positive) and coping (negative) were the most predictive motives for alcohol use, but that coping motives were uniquely predictive of alcohol-related problems even when psychological distress and alcohol use quantity/frequency were controlled. While all of these aforementioned studies are important in showing the contributions of coping motives in the alcohol-related problems pathway, none of these studies also examined impaired control over alcohol (IC) as an additional mediator of this important pathway. To our knowledge, this will be the first study to examine separate and distinct childhood trauma facets and their indirect relationships to alcohol use and alcohol-related problems through coping motives and, in turn, impaired control over alcohol. Individuals with trauma histories, both with and without PTSD diagnoses, have consistently demonstrated higher rates of coping motives for drinking, presumably to manage distress caused by these negative events [47,48,49]. 

Emotional distress mediates the association between childhood emotional abuse and alcohol-related problems [5,34]. Moreover, coping motives have been found to partially explain the association between trauma-related symptoms and increased alcohol use [31,42,48]. One study recently demonstrated that coping motives were the only type of motive associated with childhood trauma in a model comparing all four motives; childhood trauma was indirectly linked to increased alcohol-related problems through coping motives [21]. Despite consistently being linked to childhood trauma, coping motives have not been able to fully explain increased alcohol use and problems among trauma-exposed populations. Much of this work has focused on either one type of childhood trauma, such as childhood sexual abuse [50,51], or by combining all types of childhood trauma into one category [21]. Limited research has evaluated different domains of childhood trauma, but thus far, has indicated that certain traumatic events may be more likely to contribute to increased coping motives. In a sample of college students, coping motives were correlated with childhood abuse but not neglect [52]. Interestingly, Canning et al. [28] found that higher coping motives were directly related to more impaired control. However, to date, no one has examined whether both coping motives and IC serve as mediating mechanisms for distinct childhood trauma facets (i.e., emotional, physical, and sexual abuse, as well as physical neglect) in alcohol use and problems pathways. 

### 1.3. Why We Should Study Impaired Control with Coping Motives Rather Than Just Impulsivity

Ajzen’s [53] Theory of Planned Behavior states that behavioral intention is the most proximal and strongest determinant of human behavior. Yet, addiction researchers have been obsessed with construct(s) of behaviors that reflect behavioral under-control and yet lack behavioral intention, such as impulsivity, for decades. Impulsivity is “a predisposition toward rapid, unplanned reactions to internal or external stimuli without regard to the negative consequences” [54] (p. 1784). Historically, the concept of impulsivity has dominated psychological theory and research pertaining to AUDs, with over 3000 articles on the topic found in library search engines such as PsycINFO. General impulsivity is important and is associated with all forms of childhood maltreatment, especially emotional abuse [55]. Evans and Reed [32] found that women with a history of childhood sexual abuse tend to be more impulsive in their responses to measures of response inhibition (GoStop task) and initiations (immediate and delayed memory tasks). Wardell et al. [47] also found facets of impulsivity to mediate the association between childhood maltreatment and alcohol use problems. Nevertheless, impulsivity fails to consider intentions for drinking beyond limits and that perhaps limits the extent to which we are able to predict harmful heavy drinking events. 

Impaired control over alcohol (IC) reflects the failure to maintain self-prescribed limits for alcohol consumption, indicated by a difficulty avoiding alcohol use, as well as a difficult time controlling drinking once it has begun [9]. According to the existing literature, IC is the earliest indicator of risk for AUD [56,57]. For instance, Leeman et al. [58] found that IC explained the growth in alcohol consequences over three years later. Moreover, behavioral impaired control (i.e., drinking beyond heavy episodic levels despite incentives to limit consumption during ad libitum) causally followed an acute stressor (i.e., Trier Social Stress Test) in a self-administration study [59]. Clearly, impaired control is a critical risk factor for developing AUDs, particularly among adolescents and young adults [17,59,60,61,62,63], yet it is understudied as a construct compared to impulsivity. Both constructs of impulsivity and impaired control reflect a lack of behavioral control. Yet, these constructs are clearly distinct; impaired control reflects a lack of adherence to self-made limits, specifically in the context of drinking [63,64,65], whereas impulsivity does not reflect any intention to limit [66]. While several studies have investigated the relationships between impulsivity and childhood trauma [32,55,67], few have explored the potential relationship between childhood maltreatment and impaired control over alcohol, which is more proximal to the actual drinking behavior. Moreover, to our knowledge, no one has ever studied the impact of childhood trauma facets on coping motives for drinking through the additional potential mediator of IC. 

Patock-Peckham and Corbin [68] have suggested that impaired control (IC) and impulsivity are related but distinct constructs, with IC being a much stronger longitudinal predictor of alcohol problems over 12 months than all the facets of impulsivity (i.e., UPPS-P; [69]) during early adulthood. They actually had impulsivity facets and impaired control compete for variance in their longitudinal model over 12 months. Patock-Peckham and Corbin [68] found that IC was a stronger predictor than not only all facets of impulsivity, but was even stronger than alcohol use itself. Impulsivity is a popular construct, but according to the Theory of Planned Behavior [53], behavioral intentions such as those found in the IC construct may better elucidate the effects of trauma on alcohol-related problems, especially when used in conjunction with coping motives. Hence, we should be studying IC as well as impulsivity, especially since several investigators have called for greater specificity concerning a lack of behavioral control constructs [70,71]. While several studies have investigated the relationships between impulsivity and childhood trauma [32,55,67], few have explored the potential relationship between childhood maltreatment and impaired control over alcohol, which is more proximal to the actual drinking behavior. This study will reflect the first ever investigation into childhood trauma facets, coping motives, and impaired control over alcohol (IC) in the pathways of both alcohol use and its related problems. 

### 1.4. Emotional Abuse, Coping Motives, and Impaired Control

Emotional abuse seems to be particularly predictive of internalizing symptoms such as PTSD and poor sleep among college students. For instance, Patock-Peckham et al. [17] found that emotional abuse was directly related to more PTSD symptoms among 835 university students, while controlling for all other facets of childhood trauma prior to the age of 12. Furthermore, Noudali et al. [16] found that emotional abuse was directly linked to poorer sleep quality (i.e., insomnia symptoms) among 941 university students while controlling for all other forms of childhood trauma, as well as depressive symptoms, prior to the age of 12. In addition, Shin et al. ([21] (See p. 4) found that emotional abuse was strongly correlated with both coping motives for alcohol (0.26***) as well as alcohol problems (0.26***). Nevertheless, Shin’s [21] mediational model lumped all the facets of childhood trauma/maltreatment together. Combining all the facets of childhood trauma together prevented Shin et al. [21] from testing the individual facets of trauma as indirect predictors of alcohol use or problems via the unique types of motives for drinking. Thus, we sought to expand upon Shin et al.’s [21] important work by further elucidating these distinct pathways from the unique facets of trauma, while still controlling for all other trauma facets in our model. 

### 1.5. Sexual Abuse, Coping Motive, and Impaired Control

Previous investigators have explored the relationships between childhood sexual abuse, coping motives and alcohol problems. Although the effects were small, there were reliable effects for both the enhancement and coping motives in mediating the indirect links between a history of childhood sexual assault and alcohol problems among women [50]. Researchers using a large sample of 1863 women from the Chicagoland area, found that the indirect links between child sexual abuse and substance use were only partially mediated by PTSD symptoms [51]. Furthermore, the use of substances to cope only partially mediated the effect of PTSD on problematic drinking [51]. Hence, it is possible that impaired control over alcohol further elucidates the relationship between childhood sexual trauma and coping motives along the pathway of alcohol use and its related problems. We hypothesized that higher levels of sexual abuse will be indirectly associated with more alcohol-related problems through both more coping motives and impaired control and, in turn, more alcohol use. 

### 1.6. Physical Neglect, Coping Motives, and Impaired Control

The Theory of Behavior Economics [72] posits that individuals who are physically neglected may rashly overconsume available reinforcers (i.e., food or alcohol) because they fear the item will fail to be presented again. Consistent with this theory, prior studies [15,16,17] have demonstrated direct links between physical neglect and perceived impaired control. For instance, physical neglect predicted more impaired control over alcohol when controlling for mindfulness, age, gender, ethnicity, and other drug use, as well as other facets of trauma [15]. In addition, physical neglect predicted more impaired control over alcohol even when controlling for PTSD, as well as other facets of childhood trauma [17]. Finally, physical neglect was directly linked to more IC even when controlling for depression, gender, insomnia, and all other facets of trauma [16]. Nevertheless, it remains unclear as to whether or not physical neglect would be directly predictive of coping motives for drinking. We felt that we should test this potential pathway because Canning et al. [28] did find a direct link between coping motives for drinking and more IC. However, to date, no one has examined whether coping motives and IC serve as mediating mechanisms in conjunction with the distinct childhood trauma facets (i.e., emotional, physical, and sexual abuse, as well as physical neglect) on alcohol use and its related problems. Moreover, it is unclear whether or not coping motives mediate the link between physical neglect and perceived impaired control over alcohol, as evidence seems mixed [28,52]. 

### 1.7. Hypotheses

Consistent with SMH [2,3,4], we hypothesized that all forms of childhood trauma (physical, emotional, and sexual abuse, as well as physical neglect) would be linked to increased coping motives. Nevertheless, based upon Shin’s [21] findings, we did expect emotional abuse to be the most highly related form of childhood trauma directly linked to coping motives. Based upon Canning et al.’s [28] findings, we hypothesized that coping motives would be directly linked to more IC. According to Behavioral Economics Theory [72], we also hypothesized that physical neglect would be directly linked to more IC [15,16,17]. Based upon the aforementioned literature and theory, we also expected that coping motives and IC would both mediate the indirect links between distinct childhood trauma facets and alcohol use and its related problems. 

## 2. Methods

### 2.1. Participants

The participants included 612 university students (270 women, 342 men). The sample was 67% White, 14% Hispanic, 10% Asian, 4% African American, 1% Native American, and 4% reported as “other”. The mean age of the sample was 20.41 (SD = 3.27). All data was collected at Arizona State University through Sona Systems; the participants were invited to join the study with an email invitation for Introductory Psychology credit for their course requirement. Filled with more than 350 different majors across the ASU campus, Introductory Psychology courses reflect a wide variety of people and interests. 

### 2.2. Measures

#### 2.2.1. Childhood Trauma Questionnaire

The Childhood Trauma Questionnaire [20] consists of 25-items that measure emotional abuse, physical abuse, sexual abuse, physical neglect, and emotionally supportive family members from childhood retrospectively. Emotional abuse consists of 5-items, including “called names by family” and “parents wished I was never born”. Physical abuse consists of 5-items, including “hit badly enough to be noticed” and “punished with hard objects”. Sexual abuse consists of 5-items, including “was molested” and “hurt if I didn’t do something sexual”. Physical neglect consists of 5-items, including “wore dirty cloths” and “parents were drunk or high”. An emotionally supportive family consists of 5-items, including “Made me feel important” and “I felt loved”. Responses were measured on a 1–5 Likert scale with 1 = ‘never true’ to 5 = very often true. The α reliabilities among the five scales were emotional abuse 0.81, physical abuse 0.82, sexual abuse 0.92, physical neglect 0.74, and emotionally supportive family members 0.88.

#### 2.2.2. Coping Motives

Of the four-factor model of Drinking Motives by Cooper [27], we utilized solely the Coping Motives scale. There were 5-items measuring the Coping Motives. Responses included 1 = almost never/never, 2 = some of the time, 3 = half of the time, 4 = most of the time, 5 = almost always/always. The 5-items reflecting Coping Motives included “To forget your worries”, “To forget about your problems”, “Because it helps you when you feel depressed or nervous”, “To cheer up when you are in a bad mood”, and “Because you feel more self-confident and sure of yourself”. The alpha reliability for the coping subscale for this sample was 0.88. 

#### 2.2.3. Impaired Control over Alcohol

This scale reflects 10 items from the Impaired Control Scale [9]. Higher scores on this measure are reflective of a lack of perceived control over drinking (i.e., an inability to stop drinking at will). A sample item included “Even if I intended having only one or two drinks, I would end up having many more”. Responses for the Impaired Control Scale were 1 = strongly disagree, 2 = disagree, 3 = neither agree or disagree, 4 = agree, 5 = strongly agree. The α reliability for this sample was 0.82. 

#### 2.2.4. Alcohol Use (Quantity/Frequency Measure)

We combined the alcohol use quantity as well as the alcohol frequency items into a single Quantity/Frequency Scale. We converted the frequency levels into equivalent occasions per month, which ranged from 1 = 0.5 times per month to 7 = 28 times per month. The quantity levels were converted into equivalent grams of alcohol, which ranged from 1 = 10 g a month to 5 = 70 g a month. We multiplied these values and then transformed the distribution of scores through a log10 transformation [73]. 

#### 2.2.5. Alcohol Problems

The Young Adult Alcohol Problems Screening Test (YAAPST; [74]) comprises 27 items that target college students and the negative consequences associated with alcohol use within the past year. Some sample items included “Has drinking ever gotten you into sexual situations which you later regretted?”, “Have you felt sick to your stomach or thrown up after drinking?”, and “Have you ever gotten into trouble at work or school because of drinking?” The α reliability for this sample was 0.88.

### 2.3. Statistical Analysis

Using Mplus 8.3 [75], a structural equation model was evaluated with chi-square statistics, RMSEA [76,77], and CFI [78]. As our overall structural invariance test did not exceed the critical value at *p* < 0.05 when all paths were constrained to equality, it suggested that men and women did not need to be modeled separately. However, we included gender as a covariate to control for the fact that men typically consume more alcohol on average than women do [79]. Mediational effects were examined utilizing the parametric bootstrapped (k = 20,000) 90–99% asymmetric confidence interval technique for the estimates of the indirect effects (i.e., zero is not found in the interval of a mediated effect; [80,81]. 

## 3. Results 

We fit a path model with Mplus version 8.3. Our model fit the data well with χ^2^ (6df) 7.979, *p* = 0.2397; RMSEA = 0.023; 90% CI. [0.00, 0.061]; CFI = 0.999; TLI. = 0.992. Table 1 presents descriptive statistics. To interpret gender, men were coded as ones and women as zeros. Thus, a positive correlation with gender reflects a stronger relationship with being a man.

**Figure 2:**
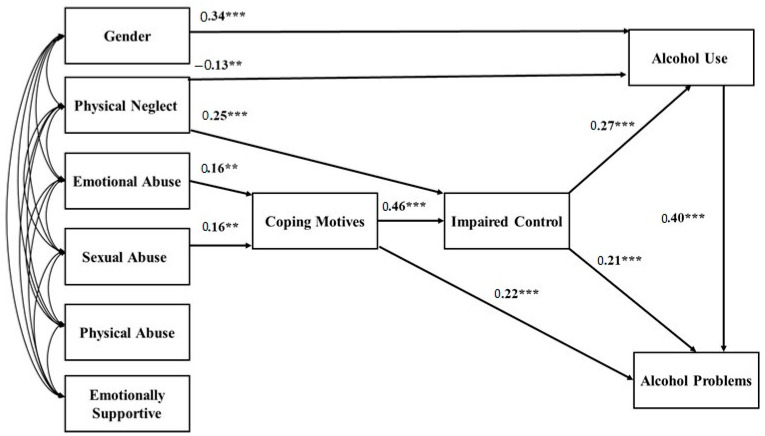
Fit path model for 612 university students. Standardized coefficients are shown with all exogenous variables allowed to correlate freely in the model. Women were coded as zeros, while men were coded as ones. Hence, a positive relationship between gender and alcohol use means that being a man was associated with more alcohol use. ** *p* < 0.01; *** *p* < 0.001.

Table 2 presents each variable name, number of complete cases out of 612, range of responses, skew, skew standard error, kurtosis, and the kurtosis standard error. Presented in Figure 2 are the standardized coefficients for the path model we tested. Table 3 presents the two, three, and four path-mediated effects we examined. 

## 4. Discussion

### 4.1. Emotional and Sexual Abuse’s Associations with Coping Motives

The Self-Medication Hypothesis [3] posits that individuals who experience painful emotions may use alcohol and other substances to cope with their negative feelings. The association between childhood trauma and AUDs has long been explored and empirically supported [6,14,18,83]. We are consistent with Canning et al. [28], who found that higher levels of coping motives directly predicted more perceived impaired control over alcohol (IC). Nevertheless, our study is novel in that it explored how specific facets of childhood trauma relate to coping motives for drinking simultaneously in conjunction with IC, which is a possible additional mediator. As hypothesized, both coping motives, as well as the perceived IC, acted as multiple mediating mechanisms in the pathways from sexual and emotional abuse to alcohol use and its related problems. However, our findings did not support our hypothesis that all forms of childhood trauma would follow this same pathway. We did not find this same pattern with physical abuse, a lack of an emotionally supportive family, or regarding physical neglect. To our knowledge, we are the first to find these specific and unique patterns of relationships for sexual and emotional abuse. The present findings are consistent with extant research showing that both sexual abuse and emotional abuse are indirectly associated with alcohol use and its related disorders [16,17]. The previous literature [21,52] has also found that coping motives for drinking mediate the pathways from childhood maltreatment to alcohol use and related problems. Nevertheless, our study is unique because we used multiple types of childhood trauma as predictors in our model, thus we were able to compare the impact of each type of trauma over and above each other form of trauma. According to the Self-Medication Hypothesis, the direct link between sexual and emotional abuse and coping motives suggests that individuals who have experienced these particular maltreatment facets drink alcohol to alleviate trauma-related emotional pain for a brief time [5,34,84]. We expanded this important existing literature by adding impaired control (i.e., perceived dysregulated drinking) as an additional mediator. 

Still, the question that then arises is why these particular subtypes of trauma (sexual and emotional abuse) demonstrate a significant association with coping motives. When compared to other forms of childhood abuse, some studies [85,86,87] have found emotional abuse to be associated with higher rates of depression and other mood disorders, a higher severity of psychiatric symptoms (i.e., hopelessness, suicidal ideality, anxiety, and impulsivity), higher emotional dysregulation, and more interpersonal problems. Other studies [88,89] revealed associations between childhood sexual abuse and similar negative outcomes, including an increased likelihood of depression, anxiety, higher levels of symptoms such as worry and loneliness, and a low quality of life. All of the aforementioned negative symptoms associated with both emotional abuse and sexual abuse may serve as potential motivators for an individual to use drinking alcohol as a coping mechanism and as a means of self-regulation [3]; this offers a possible explanation for the direct link between emotional and sexual abuse and coping motives. However, before drawing strong conclusions regarding this pattern of relationships, further longitudinal research concerning our findings seems wise. 

### 4.2. Coping Motives and Impaired Control over Drinking Alcohol

While previous research [21,52] has identified that coping motives are a mediator between childhood trauma and alcohol use, our findings are novel in that they also demonstrate a direct association between childhood trauma, coping motives, and impaired control over drinking along the pathway of alcohol use and its related problems. Our present study and the existing literature [90,91,92] have established an association between internalizing behaviors and alcohol use disorders. Theoretically supported by the Self-Medication Hypothesis [3], the internalizing pathway to alcohol-related problems suggests that experiencing affective symptoms in early childhood increases the risk of developing AUDs when individuals drink alcohol to cope with distress. Conversely, the previous literature has also established an association between externalizing behaviors [60,61,63,64,65] that is also present in our study, showing that impaired control over drinking was significantly related to alcohol use and its related problems. Consistent with the findings of Canning and colleagues [28], the direct path from coping motives to impaired control, and from impaired control to alcohol use and its related problems demonstrates an internalizing to externalizing pathway to alcohol-related consequences. This association is important because it expands our current understanding of the proposed internalizing and externalizing pathways [3,90,91,92] to alcohol-use disorders. The risks associated with the development of internalizing symptoms in early childhood [90,91,92], when joined with the risks related to IC in college-aged students [16,17,59,60,61,63], creates a subset of college-aged students who are especially vulnerable to suffering alcohol-related consequences. 

### 4.3. Physical Neglect and Impaired Control over Drinking

Our findings are highly consistent with the Behavioral Economics Theory [72] in relation to alcohol use and related problems. Behavior Economics indicates that when rewarding resources are not consistently available, children who are accustomed to physical neglect may be much more likely to consume the rewarding resources in excess when they are available. We replicated findings from the recent literature [15,16,17] that demonstrated a direct pathway from physical neglect to an impaired control over alcohol. Physical neglect is an understudied facet of childhood maltreatment. Yet, it is clear that higher levels of physical neglect predicted increased levels of impaired control over alcohol. Consistent with Goldstein [52], we demonstrate that physical neglect is not related to coping motives and that even when controlling for coping motives, physical neglect is directly linked to dysregulated drinking (i.e., impaired control). 

### 4.4. Limitations and Future Direction

While this study adds several crucial findings to the existing body of literature regarding childhood trauma, coping motives, and impaired control over alcohol, it is not without limitations. The first limitation to consider is the cross-sectional nature of these data. With cross-sectional data, we are unable to draw causal conclusions between our variables and can only explore associations. Secondly, college-aged students comprised the vast majority of our sample, limiting our generalizability. The college-aged drinking sample could potentially limit the scope of our findings because people who experience severe childhood physical abuse are less likely to attend college [93]. To address both the limits of cross-sectional data and the college-aged drinking sample, it is necessary to further explore these relationships using multiple-wave methods of study, as well as use a more community-based sample for future investigations. Another clear limitation of this current study is that we failed to include measures of emotional dysregulation in our model. Future investigators are encouraged to include measures of facets of emotional dysregulation to further map out indirect pathways to alcohol use and its related problems via childhood trauma facets. Examining emotional regulation facets as other potential mediators of drinking motives and an impaired control over alcohol seems prudent. This study is also limited in that it cannot present data regarding a community estimate of alcohol misuse and dependence among adult survivors of childhood trauma. Hence, this present study should only be viewed as an investigation of alcohol use and its related problems found among a college student sample. Future investigations should explore more community samples regarding these research questions because childhood trauma is likely to be more prevalent among ordinary citizens rather than university students.

Despite these limitations, the present study is the first to demonstrate a mediating pathway between sexual and emotional abuse, coping motives, impaired control, and alcohol use and its related problems. These distinctive pathways are crucial because they suggest that individuals who have suffered emotional or sexual abuse may be at an increased risk of increased alcohol use and its related problems. This may be especially true when the motive for alcohol use is to cope with trauma-associated internalizing symptoms and emotions. Thus, our study suggests that both coping motives and impaired control may be good therapeutic targets of intervention for individuals who have experienced either sexual or emotional abuse traumas prior to the age of 12. Our findings are novel in that we found that both coping motives and impaired control acted as multiple mediating mechanisms between sexual and emotional abuse in alcohol use and its related problems. We are much less certain regarding what the therapeutic target should be in order to disrupt the link between physical neglect and an impaired control over alcohol outside, perhaps, behavioral protective strategy training. Nevertheless, we hope that future investigators will further continue to explore the physical neglect to dysregulated drinking (i.e., impaired control) link in the quest to reduce AUDs. 

## Figures and Tables

**Table 1:** Means, standard deviations, and correlations among all variables in the path model.

M	SD	Measures	1	2	3	4	5	6	7	8	9	10
0.56	0.49	1. Gender	**1.00**									
1.45	0.62	2. Physical Neglect	0.17	**1.00**								
1.64	0.79	3. Emotional Abuse	−0.07	0.47	**1.00**							
1.30	0.74	4. Sexual Abuse	0.04	0.42	0.42	**1.00**						
1.44	0.69	5. Physical Abuse	0.09	0.51	0.68	0.46	**1.00**					
3.82	0.96	6. Emotionally Supportive	−0.03	−0.06	−0.54	−0.28	−0.36	**1.00**				
2.04	0.94	7. Coping Motives	−0.03	0.25	0.31	0.28	0.25	−0.25	**1.00**			
1.78	0.67	8. Impaired Control	0.03	0.29	0.17	0.19	0.17	−0.25	0.49	**1.00**		
2.01	0.62	9. Alcohol Use	0.14	0.01	0.02	0.00	−0.02	0.04	0.35	0.36	**1.00**	
0.67	0.62	10. Alcohol Problems	0.09	0.23	0.21	0.21	0.20	−0.14	0.51	0.49	0.56	**1.00**

Means, standard deviations, and correlations among all variables based on a sample of 612 individuals (342 men & 270 women).

**Table 2:** Variable name, number of complete cases out of 612, range of responses, skew, skew standard error, kurtosis, kurtosis standard error.

Variable	Complete Cases	Range	Skew	S.E.	Kurtosis	S.E.
Gender	612	(0.00, 1.00)	−0.240	0.099	−1.941	0.197
Physical Neglect	593	(1.00, 3.80)	1.438	0.100	1.223	0.200
Emotional Abuse	593	(1.00, 5.00)	1.583	0.100	2.318	0.200
Sexual Abuse	593	(1.00, 5.00)	2.734	0.100	7.083	0.200
Physical Abuse	594	(1.00, 5.00)	2.132	0.100	4.640	0.200
Emotionally Supportive Family	592	(1.00, 5.00)	−0.796	0.100	0.094	0.201
Coping Motives for Drinking	585	(1.00, 5.00)	0.916	0.101	0.190	0.202
Impaired Control Over Alcohol (IC)	610	(1.00, 4.10)	0.760	0.099	−0.121	0.198
Alcohol Use Quantity/Frequency	610	(0.70, 3.29)	−0.418	0.099	−0.478	0.178
Alcohol-Related Problems	579	(0.00, 4.37)	1.781	0.102	4.894	0.203

Note: We had complete data for 94.61% of our sample of 612 cases (270 women; 342 men). Due to data collection occurring with the Qualtrics online survey tool, we did not experience extreme outliers for any of our measures. Moreover, our measures of skew and kurtosis suggest that our variables are in the acceptable range for normality in our path model. Brown [82] suggests that one has acceptable values for skew if they do not exceed + or − 3.00, and that kurtosis values are acceptable if they do not exceed + or − 10.00.

**Table 3:** Mediated pathways.

Pathway Effects	Indirect Effect	Z-Score	*p*-Value	95%CI
Impaired Control (IC)				
Sexual Abuse→Coping Motives→IC	0.066	2.795	0.005	(0.022, 0.116)
Emotional Abuse→Coping Motives→IC	0.060	2.164	0.030	(0.006, 0.114)
Alcohol Use				
Coping Motives→IC→Alcohol Use	0.081	5.349	<0.001	(0.054, 0.113)
Physical Neglect→IC→Alcohol Use	0.045	2.730	0.006	(0.017, 0.083)
Sexual Abuse→Coping Motives→IC→Alcohol Use	0.017	2.476	0.013	(0.006, 0.033)
Emotional Abuse→Coping Motives→IC→Alcohol Use	0.015	2.005	0.045	(0.002, 0.032)
Alcohol-Related Problems (ARP)				
Coping Motives→IC→ARP	0.061	4.048	<0.001	(0.034, 0.094)
Sexual Abuse→Coping Motives→ARP	0.029	2.423	0.015	(0.010, 0.060)
Emotional Abuse→Coping Motives→ARP	0.027	1.957	0.050	(0.004, 0.058)
Physical Neglect→IC→ARP	0.034	2.552	0.011	(0.012, 0.066)
Coping Motives→IC→Alcohol Use→ARP	0.032	5.201	<0.001	(0.022, 0.046)
Sexual Abuse→Coping Motives→IC→ARP	0.013	2.381	0.017	(0.004, 0.026)
Emotional Abuse→Coping Motives→IC→ARP	0.011	1.854	0.064	(0.002, 0.026)
Sexual Abuse→Coping Motives→IC→Alcohol Use→ARP	0.007	2.454	0.014	(0.002, 0.013)
Emotional Abuse→Coping Motives→IC→Alcohol Use→ARP	0.006	1.971	0.049	(0.001, 0.013)

## Data Availability

Data availability upon request of the corresponding author.
